# Polycomb proteins translate histone methylation to chromatin folding

**DOI:** 10.1016/j.jbc.2023.105080

**Published:** 2023-07-25

**Authors:** Ludvig Lizana, Negar Nahali, Yuri B. Schwartz

**Affiliations:** 1 Department of Physics, Integrated Science Lab, Umeå University, Umeå, Sweden; 2 Department of Informatics, Centre for Bioinformatics, University of Oslo, Oslo, Norway; 3 Department of Molecular Biology, Umeå University, Umeå, Sweden

**Keywords:** epigenetics, polycomb, chromatin structure, histone methylation, *Drosophila*

## Abstract

Epigenetic repression often involves covalent histone modifications. Yet, how the presence of a histone mark translates into changes in chromatin structure that ultimately benefits the repression is largely unclear. Polycomb group proteins comprise a family of evolutionarily conserved epigenetic repressors. They act as multi-subunit complexes one of which tri-methylates histone H3 at Lysine 27 (H3K27). Here we describe a novel Monte Carlo–Molecular Dynamics simulation framework, which we employed to discover that stochastic interaction of Polycomb Repressive Complex 1 (PRC1) with tri-methylated H3K27 is sufficient to fold the methylated chromatin. Unexpectedly, such chromatin folding leads to spatial clustering of the DNA elements bound by PRC1. Our results provide further insight into mechanisms of epigenetic repression and the process of chromatin folding in response to histone methylation.

Epigenetic repression often involves covalent histone modifications. In many instances, we know which proteins install the modifications and specifically recognize them. Yet, how the presence and recognition of a histone modification translate into changes in chromatin structure that benefit the repression is largely unclear. Epigenetic repression of developmental genes by the Polycomb system is essential for all multicellular animals (1, 2). It involves tri-methylation of histone H3 at Lysine 27 (H3K27) by Polycomb Repressive Complex 2 (PRC2) (3, 4) and binding of the modified histone by Polycomb Repressive Complex 1 (PRC1) (5). In fruit flies *Drosophila melanogaster*, the only organism where this was directly tested, H3K27 methylation is required for the repression (6). It appears to act as the molecular mark assuring that both copies of a target gene remain repressed after the DNA replication (7, 8).

Mechanisms by which Polycomb complexes repress transcription are not well understood but seem to involve modifications of the chromatin structure. The chromatin of genes repressed by Polycomb complexes (hereafter Polycomb-repressed genes) is folded in an unusual way. It is more compact compared to chromatin of regular inactive and transcriptionally active genes and displays higher degree of intermixing (9). This chromatin structure requires PRC1 and was suggested to involve self-interactions of one of its subunits (9). Whether tri-methylation of H3K27 is implicated in the chromatin folding has not been investigated.

*Drosophila* genes regulated by the Polycomb system contain specialized Polycomb Response DNA Elements (PREs), which serve as high-affinity binding sites for PRC1 and PRC2 (10). Polycomb-repressed genes are embedded in broad chromatin domains enriched in tri-methylated H3K27 (11, 12, 13). Despite this, PREs remain the only sites where PRC1 is stably bound. Together with the observation that PRC1 continues to bind PREs in cells deprived of PRC2 (14), this discounts the hypothesis that methylation of H3K27 is used to mark genes for PRC1 recruitment. If tri-methylated H3K27 does not serve to recruit PRC1 to genes, what is this epigenetic mark good for?

It is tempting to hypothesize that the trimethylation of H3K27 is part of the chromatin folding mechanism and thereby epigenetically marks Polycomb-repressed genes for folding. Testing this hypothesis experimentally is challenging for two main reasons. First, there are many proteins involved, which makes the *in vitro* reconstitution of a Polycomb-repressed gene prohibitively difficult. Second, complex biochemical interactions between PRC1 and PRC2 (15, 16) make *in vivo* genetic knock-out experiments hard to interpret. To circumvent this problem, we took a computational approach and developed a novel Monte Carlo–Molecular Dynamics (MC-MD) simulation framework. The framework uses Large-scale Atomic/Molecular Massively Parallel Simulator (LAMMPS) (17) to model the chromatin motion explicitly but treats the binding of PRC1 to PREs probabilistically. This approach has three advantages over the explicit simulation of the entire system. First, it requires no assumptions regarding the physical properties of PRC1 in the nucleoplasm. Second, it does not rely on the prior knowledge of PRC1 affinity to PREs, which, so far, has not been directly measured. Third, there is a considerable computational gain for not tracking PRC1 complexes unbound to chromatin.

## Results

**Figure 1:**
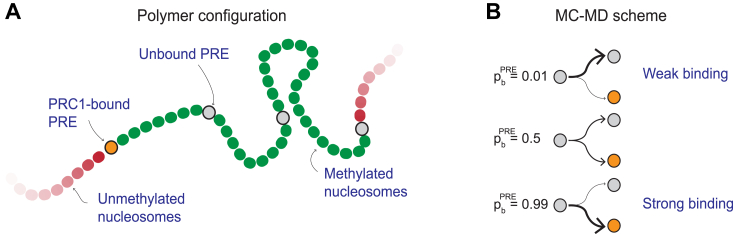
**Polymer configuration.***A*, polymer model. We model chromatin as a semi-flexible polymer with individual units corresponding to nucleosomes or PREs. PREs may be PRC1-bound (*orange*) or free (*gray*). If bound, PREs have an affinity to nucleosomes tri-methylated at H3K27 (*green*). Neither PRC1-bound nor free PREs adhere to unmethylated nucleosomes (*red*). *B*, Monte-Carlo–Molecular Dynamics (MC-MD) protocol. Before the polymer MD simulations (using LAMMPS), we probabilistically assign PREs to the PRC1-bound state. The probability of this event (*i.e.*, PRC1 binding probability $p_{b}^{PRE}$) decreases with increasing dissociation constant $K_{D}^{PRE}$.

To build the model, we represent the chromatin as a semi-flexible self-avoiding polymer fiber with repeating units (monomers) of 10-nm (nucleosome) size (denoted σ). The polymer contains 360 monomers of four possible types (Fig. 1*A*): nucleosomes containing H3K27me3 (methylated nucleosomes, green), nucleosomes lacking H3K27me3 (unmethylated nucleosomes, red), PRC1-bound PREs (orange), and unbound PREs (light grey). A Polycomb-repressed locus is located in the center of the polymer and is represented as a stretch of methylated nucleosomes with four embedded PREs (see Experimental procedures for more details).

In the first modeling step, we take 150 polymer fibers, organized as described above but with all PREs unbound by PRC1, and simulate their movements with LAMMPS until they reach thermodynamic equilibrium. We then populate the PREs of each equilibrated polymer with PRC1 complexes based on the binding probability $p_{b}^{PRE}$ (Fig. 1*B*). To this effect, we draw uniform random numbers r∈(0,1) and designate each PRE as PRC1-bound if $r<p_{b}^{PRE}$. This is followed by a round of LAMMPS simulations that capture events during characteristic residence time (100 s) of PRC1 on chromatin (18). In this simulation round, PRC1-bound PREs are attracted to methylated nucleosomes. If a PRC1-bound PRE and a methylated nucleosome come close, chromatin loops may form.

**Figure 2:**
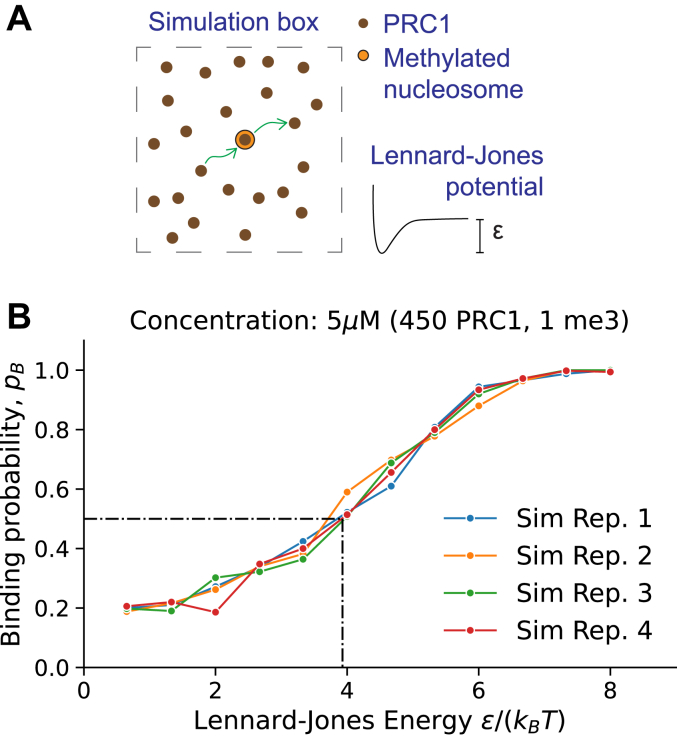
**Simulated binding probability (bound fraction) across Lennard-Jones energies**ε**.***A*, simulation setup where one binding site (*orange*) is surrounded by diffusing particles (*brown*). This setup resembles PRC1 binding to H3K27me3 peptides studied experimentally in (5). *B*, calibration curve. Each line (Rep. 1–4) shows four different simulation results with the same parameters (450 particles, 1 binding site). The *dots* represent the fraction of times the H3K27me3 binding site is bound by at least one PRC1 particle over 500 uncorrelated time points. The simulation parameters are at $K_{D}$ conditions when the $p_{b}=0.5$. That is, $\varepsilon /\left(k_{B}T\right)=3.94$ (*dash-dotted line*).

We model the attraction between PRC1-bound PREs and H3K27me3 using the Lennard-Jones potential. The potential requires calibration to recapitulate the binding affinity between PRC1 and H3K27me3. Two independent approaches were used for this purpose. First, we ran a series of designated many-particle simulations using a range of Lennard-Jones parameters to select the one at which the fraction of bound PRC1-H3K27me3 particles matched the dissociation constant ($K_{D}^{H3K27me3}$) measured experimentally (Fig. 2). Second, we used methods of statistical mechanics to derive the equation linking the Lennard-Jones potential to the dissociation constant. Below we describe the two approaches in detail.

To find appropriate Lennard-Jones potential using LAMMPS, we simulated a fixed concentration of diffusing particles and one binding site (Fig. 2*A*). Then we varied the Lennard-Jones parameter ε and tracked the fraction of bound proteins – this is a proxy for the binding probability $p_{b}$ – over several simulation runs. The simulations are at $K_{D}$ conditions when $p_{b}=0.5$. Figure 2*B* shows the calibration data. Each line corresponds to four separate simulations (’replicates’) having the same particle concentration c=5μM. To get good statistics, we used a larger simulation box than in our polymer simulations and thus more particles to achieve the desired concentration (450 particles and $V={\left(52\sigma \right)}^{3}$). The dots in each line represent an average of 500 simulated data points. Having four replicates (Rep. 1–4) amounts to 2000 data points for each value of ε. To get the numerical estimate for the $K_{D}=5\mu M$ condition, we draw a horizontal line at $p_{b}=0.5$. We find that it intersects with the $p_{b}$ curves when $\varepsilon /\left(k_{B}T\right)=3.94$.

Instead of doing simulations, we may estimate $K_{D}$ theoretically, using statistical mechanics, in terms of ε assuming that the simulation volume is large. We derive this estimate in three steps. First, we calculate the binding energy $E_{b}$ in terms of the monomer attraction. By definition, $E_{b}$ is the energy it takes to move a test particle from the bottom of the energy well past the interaction distance. Because the monomer attraction is constant beyond this distance (zero in our notation), moving the test particle even further is not associated with any extra energy cost. Using Equation 5 (see Polymer modeling part of Experimental procedures), $E_{b}$ is expressed in terms of the distance-dependent monomer attraction as:

(14) $$E_{b}=V_{bind}\left(r_{\min}\right)-V_{bind}\left(r_{\mathrm{int}}\right)\text{.}$$

Using that $r_{\min}=2^{1/6}\sigma$ and $r_{\mathrm{int}}=2.5\sigma$, we find that

(15) $$E_{b}\approx -0.938\varepsilon \text{.}$$

In the second step, we consider the binding probability $p_{b}$. It combines the binding energy $E_{b}$ and entropy cost from bringing a test particle near the binding site. This cost grows with the volume V. From statistical mechanics (19), we have $p_{b}={\left(1+\exp\left(E_{b}/\left(k_{B}T\right)\right)/V\right)}^{-1}$. However, if there is a concentration c of particles, this expression modifies to

(16) $$p_{b}=\frac{c}{c+c_{0}e^{E_{b}/\left(k_{B}T\right)}}\text{,}$$

where $c_{0}$ is standard state. This state is often set to 1 M. However, in our simulations $c_{0}=1.67\times 10^{-3}$ M. We derive this value as follows. The concentration of one 10 nm particle in the simulation volume $V={\left(23\times 10\;nm\right)}^{3}$ is 0.12μM. Thus, the concentration of a filled volume with $24^{3}$ particles is $c_{0}=0.12\;\mu M\times 24^{3}=1.67$ mM.

Lastly, we relate $E_{b}$ to $K_{D}$ *via* $p_{b}$. From first-order chemical kinetics, one can show that (see Experimental procedures for details)

(17) $$p_{b}=\frac{c}{c+K_{D}}$$

Comparing expressions (16, 17), we identify that the binding constant is

(18) $$K_{D}=c_{0}e^{E_{b}/\left(k_{B}T\right)}\approx c_{0}e^{-0.938\varepsilon /\left(k_{B}T\right)}$$

where we used Equation 15. in the last step. Finally, rewriting this equation gives

(19) $$\frac{\varepsilon }{k_{B}T}\approx -\frac{1}{0.938}\ln\left(\frac{K_{D}}{c_{0}}\right)$$

This equation allows us to calculate the Lennard-Jones parameter ε associated with a desired $K_{D}$. For example, if $K_{D}=5\mu M$ and $V={\left(23\sigma \right)}^{3}$, then $\varepsilon =6.20k_{B}T$.

The two approaches to calibrate the Lennard-Jones parameter yielded qualitatively similar albeit not identical results. While both approaches have merit, they are not perfect representations of the actual PRC1-H3K27me3 system. For example, in the numerical simulations, one H3K27me3 ’particle’ may attract more than one PRC1 ’particle’. While this is not inconceivable, it has not been observed in experiments. This contrasts with the theoretical estimate that forbids multiple binding but, on the other hand, assumes that all proteins have the same size. Because both approaches have limitations, the average of the two estimates:

(20) $$\frac{\varepsilon }{k_{B}T}=\frac{3.94+6.20}{2}=5.07\approx 5.1$$

was used for further analyses.

### Interactions between PRE-anchored PRC1 and tri-methylated H3K27 fold the chromatin

**Figure 3:**
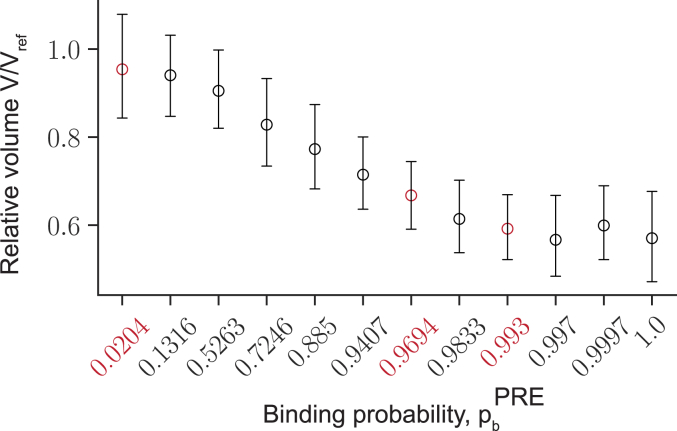
**Interactions between PRE-anchored PRC1 and tri-methylated H3K27 reduce the volume of methylated chromatin.** The average simulated volume of the Polycomb-repressed locus at varying $p_{b}^{PRE}$ relative to a reference case lacking PREs ($V_{ref}$). The error bars indicate the 95% confidence interval and the *circles* show the means.

**Figure 4:**
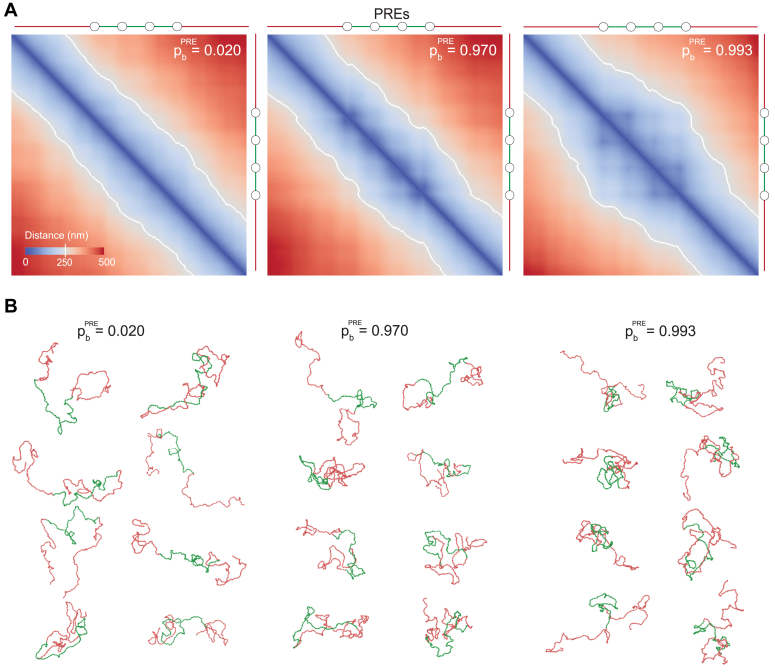
**Pairwise interactions between PRE-anchored PRC1 and tri-methylated H3K27 fold the chromatin.***A*, heat-map representation of pairwise distances between monomers for three $p_{b}^{PRE}$ values. Note that at high $p_{b}^{PRE}$ the distance heat-maps display the off-diagonal spots suggesting that PREs come in closer spatial proximity more often than other monomers located at similar linear distances. PRE positions are indicated as empty circles next to heat-maps. *B*, snapshots of representative simulated polymer 3D configurations.

To understand whether contacts between PRC1-bound PREs and methylated nucleosomes may lead to excessive chromatin folding of the Polycomb-repressed locus, we run the simulations at different $p_{b}^{PRE}$ and calculated the volume of the central part of the polymer $V=4\pi R_{g}^{3}/3$, using the Radii of Gyration ($R_{g}$) as a proxy for the polymer’s size. The Radius of Gyration is defined as the root mean square distance from the polymer’s center of mass to all monomers [Equation 9]. Plotting V in relation to the average volume of a polymer lacking PREs ($V_{ref}=V\left(p_{b}^{PRE}=0\right)$) reveals that the volume of the Polycomb-repressed locus shrinks with increasing $p_{b}^{PRE}$ eventually dropping to approximately half of the initial unfolded state (Fig. 3). The extent of shrinking agrees well with super-resolution microscopy measurements, which indicate that the chromatin of the Polycomb-repressed genes has 40 to 60% lower volume compared to that of the transcriptionally inactive genes not subjected to the repression (9). Consistently, the heat-map representations of the average pairwise distances between all monomers indicate that those within the Polycomb-repressed locus become shorter as $p_{b}^{PRE}$ grows (Fig. 4*A*). The central part of the polymer does not fold if it contains PREs but lacks methylated nucleosomes or has methylated nucleosomes but lacks PREs (Fig. S1). The snapshots of individual polymer fibers confirm the folding trend but also indicate that there is considerable variation between individual fibers even at high $p_{b}^{PRE}$ (Fig. 4*B*).

To summarize, when PREs are bound by PRC1 most of the time, the interactions of the PRE-anchored PRC1 with tri-methylated H3K27 fold the surrounding chromatin. Within the range of the binding probabilities $p_{b}^{PRE}\approx 0.5-0.95$, the degree of chromatin folding changes significantly in response to small $p_{b}^{PRE}$ fluctuations. How does this range relate to the probability of PRC1 binding to PREs inside the *Drosophila* cell nucleus? The probability depends on the PRC1 concentration ($c_{PRC1}$) and the strength of PRE-PRC1 binding reflected by the dissociation constant ($K_{D}^{PRE}$). Assuming that the number of PRC1 complexes in the nucleus is substantially higher than the number of PREs (for experimental support of the assumption see: (13, 14, 20, 21, 22)), we can view the binding of PRC1 to PREs as pseudo first-order reaction [PRE]+[PRC1]↔[PRE:PRC1] and express the binding probability as:

(21) $$p_{b}^{PRE}=\frac{c_{PRC1}}{c_{PRC1}+K_{D}^{PRE}}$$

See Experimental procedures for the derivation.

Two independent studies estimated $c_{PRC1}$ in *Drosophila* embryonic nuclei as 0.1 to 0.3 μ M (20, 22). Using the average value of $c_{PRC1}=0.2$ μ M in Equation 21, we see that the binding probability $p_{b}^{PRE}=0.5$ requires PREs to bind PRC1 with $K_{D}^{PRE}=200$ nM, while the $p_{b}^{PRE}=0.95$ corresponds to $K_{D}^{PRE}=10$ nM. Put another way, compared to the interaction with H3K27me3 ($K_{D}^{H3K27me3}∼5$ μ M) (Fischle *et al.* 2003), PRC1 ought to bind PREs 25 to 500 times stronger. We are not aware of an experimental method to measure $K_{D}^{PRE}$ directly. Nevertheless, the difference in the PRC1 binding at PREs and the flanking methylated chromatin measured by Chromatin Immunoprecipitation (ChIP) suggests that such stronger binding of PRC1 to PREs is feasible (Kahn *et al.* 2016). Overall, our calculations and modeling argue that, in *Drosophila* nuclei, PREs are likely to bind PRC1 most of the time and this enables the folding of the surrounding chromatin *via* interactions of the PRE-anchored PRC1 with tri-methylated H3K27.

### Broad range of PRC1 affinities toward H3K27me3 support chromatin folding

**Figure 5:**
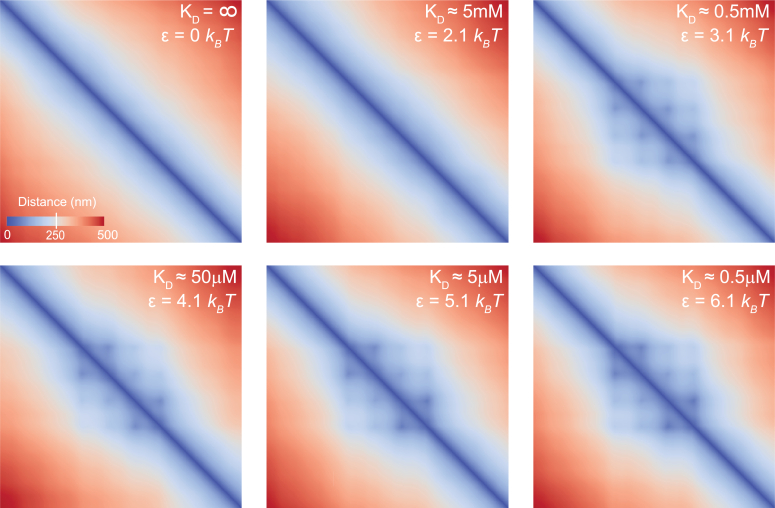
**Chromatin folding at a broad range of PRC1 affinities towards H3K27me3.** Heat-map representation of pairwise distances between monomers for six PRC1:H3K27me3 interaction energies (e). We estimate that approximately 10-fold difference in $K_{D}^{H3K27me3}$ corresponds to 1k_B_T difference in ε. The heat-map for ε = 5.1 *k*_*B*_*T* (the *middle panel* of the *bottom row*) corresponds to the $K_{D}^{H3K27me3}$ = 5 mM reported for *Drosophila* PRC1. Each heat-map represents 400 independent polymer configurations. The heat map for ε = 0 *k*_*B*_*T* (the leftmost panel of the *top row*) serves as a negative control.

Mammalian genomes encode several closely related Cbx proteins orthologous to the *Drosophila* PRC1 subunit that interacts with H3K27me3. These proteins vary in their affinities towards H3K27me3 and most of them appear to bind H3K27me3 weaker than their *Drosophila* counterpart (23, 24). We wondered to what extent the folding of methylated chromatin depends on the strength of PRC1:H3K27me3 interaction. To address this question, we performed simulations under the condition that PRC1 is always bound to PREs ($p_{b}^{PRE}=1$) but varied the attraction between PRC1 and H3K27me3 scanning the range of $K_{D}^{H3K27me3}$ from 5 mM to 0.5 μM. As illustrated by Figure 5, stochastic interactions of the PRE-anchored PRC1 with H3K27me3 fold chromatin at a broad range of $K_{D}^{H3K27me3}$. Initially, the extent of folding decays slightly as interactions weaken and appears to undergo an abrupt transition to fully unfolded state when $K_{D}^{H3K27me3}$ increases from 0.5 mM to 5 mM. The latter resembles transition from a globular compact state to fluctuating random polymer state described by the “strings and binders switch” model of chromatin folding (25). Not pursued here, it would be fascinating to investigate this resemblance further in future analyses. It may be particularly interesting to accurately define the range of PRC1:H3K27me3 interaction energies that correspond to the transition zone and investigate how the transition properties depend on the number and relative distribution of PREs.

### Chromatin folding can be re-established shortly after DNA replication

**Figure 6:**
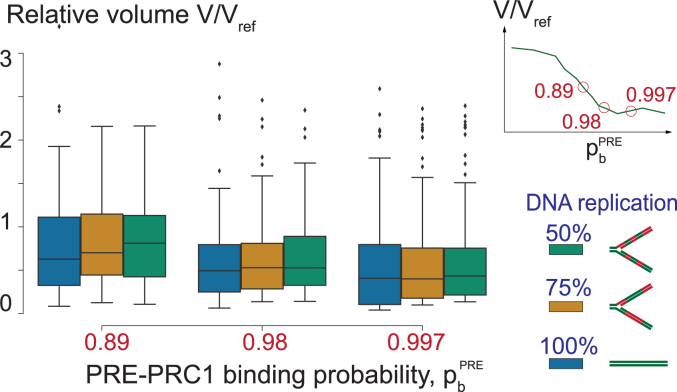
**Chromatin of Polycomb-repressed genes can be folded shortly after the DNA replication.** The boxplots show distributions of relative volumes of Polycomb-repressed loci within 150 polymers simulated at three different probabilities ($p_{b}^{PRE}$). The boxplots indicate the median and span the interquartile range. The whiskers show the lowest and the highest values excluding outliers, which are defined as values outside the 1.5 interquartile range. For each $p_{b}^{PRE}$, indicated below and on the schematic in the *upper right* corner, we randomly replaced a fraction of the H3K27me3 nucleosomes with unmethylated ones: 50% methylated nucleosomes *left* (*green*) and 75% methylated nucleosomes *left* (*orange*). The *blue boxplot* shows the variation without nucleosome replacement. Note that removing a significant fraction of methylated nucleosomes does not prevent the folding. The relative volumes are scaled to a reference case lacking PREs ($V/V_{ref}$).

The results of our simulations support the hypothesis that the tri-methylation of H3K27 labels Polycomb-repressed genes for chromatin folding. However, to be relevant for the epigenetic transmission of the repressed state, the methylation-driven folding needs to be re-established on daughter copies of a Polycomb-repressed gene shortly after DNA replication. During replication, parental H3 molecules with their posttranslational modifications are randomly partitioned between the two replicating chromatids (26, 27). The remainder is supplied *via* replication-coupled synthesis of unmodified histones. Therefore, immediately after the replication, the density of the H3K27me3-containing nucleosomes drops two-fold and is gradually restored in time for the next replication cycle. Would such dilution of the methylated nucleosomes be compatible with methylation-dependent chromatin folding? To address this question, we repeated MC-MD simulations for three different $p_{b}^{PRE}$ values that support chromatin folding. However, this time, we randomly replaced 50% and 25% of H3K27 tri-methylated nucleosomes with unmethylated ones to mimic the situations immediately after the replication and half-way through the re-acquisition of the fully methylated state. As illustrated by Figure 6, when most PREs are occupied by PRC1 ($p_{b}^{PRE}=0.98$ or 0.997), even two-fold dilution of methylated nucleosomes has no effect on chromatin folding. When PRC1 binds PREs less often, ($p_{b}^{PRE}=0.89$), 50% dilution leads to visible increase in the median relative volume of the Polycomb-repressed locus. However, the increase is not significantly different compared to the median relative volume of the fully methylated locus. Importantly, chromatin folding is restored when the density of methylated nucleosomes reaches 75%. To summarise, our simulations argue that tri-methylation of H3K27 is capable of marking Polycomb-repressed genes for epigenetic inheritance of chromatin folding.

### Chromatin folding leads to spatial clustering of PREs

Besides the overall folding of the Polycomb-repressed locus, the distance heat-maps reveal off-diagonal spots (Figs. 4 and 5). Best seen on the maps with high $p_{b}^{PRE}$ these spots suggest that, when the chromatin of the repressed locus folds, PREs end up close to each other more frequently than other monomers located at comparable linear distances. Strikingly, similar spots were noted in the contact maps of some of the Polycomb-repressed genes assayed by Chromosome Conformation Capture (Hi-C) (28, 29). Interpreted as bases of chromatin loops formed by PRE clusters, these spots were hypothesized to arise from the protein-protein interactions between PRE-anchored PRC1 complexes (28, 29). Although consistent with the biochemical properties of PRC1 subunits *in vitro*, this explanation cannot apply to our model. Our model does not explicitly simulate PRC1 and, therefore, provides no possibility for direct PRE-PRE interactions. Instead, the PRE clustering appears to have a geometric/probabilistic explanation, which we present below.

**Figure 7:**
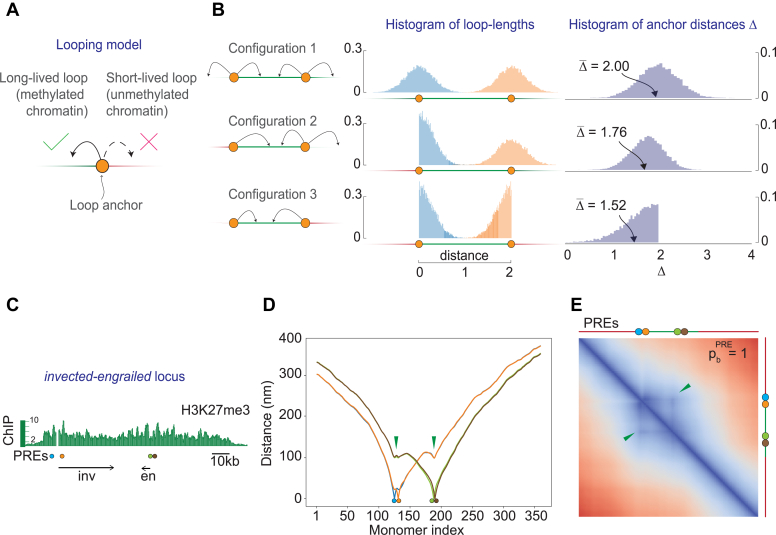
**PRE clusters emerge during chromatin folding.***A*, simple looping model. The model considers anchor points (*orange circles*) on a line that may touch surrounding line segments and form loops. Depending on the segment type, which could be either "sticky" (*green*) or "non-sticky" (*red*), the loops will be long-lived or short-lived. The model postulates that only long-lived loops are stable enough to be detected. *B*, three basic configurations of anchor points and the two chromatin types. Arrows indicate the directions in which the anchor points may form long-lived loops. Shown to the right are the corresponding histograms of the simulated loop lengths and anchor distances. The average anchor distance ($\bar{\Delta }$) depends on the relative arrangement of anchor points and the "sticky" chromatin segments. *C*, the map of the *invected-engrailed* (*inv-en*) locus repressed by the Polycomb system. The distribution of H3K27me3 ChIP signal (13) across the locus indicates the extent of methylated chromatin relative to PREs (*blue*, *orange*, *green*, and *brown circles*) and *inv* and *en* transcription units (*arrows*). *D*, color-coded curves show the average 3D distances between individual PREs (marked with *circles*) and the rest of the monomers (indexed from the *left* to the *right* edge of the polymer). The 3D distances between a PRE and other monomers increase with their separation along the polymer. However, *green arrows* point to valleys, which indicate that distances become smaller, compared with those to the preceding or the subsequent monomers, if the other monomer is also a PRE. *E*, heat-map representation of the pairwise distances between the monomers within the simulated *inv-en* locus. The *arrows* point to off-diagonal spots formed by PREs.

Let us first consider a point (*e.g.* PRC1-bound PRE) on a line that may touch surrounding line segments and form a loop (Fig. 7*A*). Depending on the segment type, which could be either "sticky" (*e.g.* H3K27 tri-methylated chromatin) or "non-sticky" (*e.g.* unmethylated chromatin), the loop will be long-lived or short-lived. The likelihood to touch a specific segment point depends on its distance from the anchor point. The exact distance dependence is not critical for our argument. Therefore, for simplicity, we assume that the loop lengths (l) follow a Gaussian distribution where $l_{0}$ denotes the loop anchor’s position and $\sigma_{l}$ denotes the standard deviation.

(22) $$g\left(l\right)=\frac{\exp\left(-{\left(l-l_{0}\right)}^{2}/\left(2\sigma_{l}^{2}\right)\right)}{\sqrt{2\pi \sigma_{l}^{2}}}$$

and say that only long-lived loops are stable enough to be detected. We then use simple stochastic simulations to model the behavior of two anchor points in three basic configurations (Fig. 7*B*).

In the first configuration, the "sticky" segments surround both anchor points. As a result, those may form stable loops to the left and right with the same probability. We put the loop anchors at positions $l_{1}=0$ and $l_{2}=2$, so that Δ=2 whenever there is no loop, and draw random loop lengths from the distributions below

(23) $$\begin{array}{rrr} g\left(l_{1}\right) & = & \frac{\exp\left(-l_{1}^{2}/\left(2\sigma_{l}^{2}\right)\right)}{\sqrt{2\pi \sigma_{l}^{2}}}\text{,}\\ g\left(l_{2}\right) & = & \frac{\exp\left(-{\left(l_{2}-2\right)}^{2}/\left(2\sigma_{l}^{2}\right)\right)}{\sqrt{2\pi \sigma_{l}^{2}}} \end{array}$$

where $\sigma_{l}=0.2$. To mimic many looping events, we draw $10^{4}$ random numbers from these distributions and calculate the absolute distance

(24) $$\Delta =\left|l_{1}-l_{2}\right|$$

As illustrated by Figure 7*B*, the histograms of resulting loop-lengths are symmetric as is the distribution of the absolute distances Δ between the two anchor points. Consistently, the average distance between the anchor points $\bar{\Delta }$ equals 2, which is identical to their linear separation because the stable loops are equally likely to form in any direction.

In the second configuration, the leftmost segment is "non-sticky", while other segments remain the same as in the first configuration. In this setup, we cannot use Equation 22 for the left anchor point as it assumes that the loops are symmetric around $l_{0}$. Instead, we use a half Gaussian. For the right anchor, we use $g\left(l_{2}\right)$ as above.

(25) $$\begin{array}{rrr} g_{\frac{1}{2}}\left(l_{1}\right) & = & \frac{\exp\left(-l_{1}^{2}/\left(2\sigma_{l}^{2}\right)\right)}{\sqrt{\pi \sigma_{l}^{2}/2}},l_{1}>0\\ g\left(l_{2}\right) & = & \frac{\exp\left(-{\left(l_{2}-2\right)}^{2}/\left(2\sigma_{l}^{2}\right)\right)}{\sqrt{2\pi \sigma_{l}^{2}}} \end{array}$$

As in this arrangement no long-lived loops form toward the leftmost segment (Fig. 7*B*, middle row), the histogram of the loop-lengths becomes asymmetric and the average distance between the anchor points shortens to $\bar{\Delta }=1.76$. In other words, compared to the first configuration, the anchor points become statistically closer.

Finally, we consider the configuration where only the segment between the two anchor points is "sticky" (Fig. 7*B*, bottom row). In this case, stable loops may form only in the direction towards the other anchor point. For this case, we draw loop lengths from two half-Gaussians

(26) $$\begin{array}{rrr} g_{\frac{1}{2}}\left(l_{1}\right) & = & \frac{\exp\left(-l_{1}^{2}/\left(2\sigma_{l}^{2}\right)\right)}{\sqrt{\pi \sigma_{l}^{2}/2}},l_{1}>0\\ g_{\frac{1}{2}}\left(l_{1}\right) & = & \frac{\exp\left(-{\left(l_{2}-2\right)}^{2}/\left(2\sigma_{l}^{2}\right)\right)}{\sqrt{\pi \sigma_{l}^{2}/2}},l_{2}<2 \end{array}$$

As a result, the average distance shortens even more to $\bar{\Delta }=1.52$. The latter argues that the propensity to cluster is the strongest for PREs located close to the edges of an H3K27me3 domain.

Interestingly, the *invected-engrailed (inv-en)* locus whose four PREs appear clustered in Hi-C experiments, has this kind of configuration (28, 29). We therefore attempted to reproduce the clustering of *inv-en* PREs in our MC-MD simulation framework. To this effect, we repositioned PRE monomers within the simulated polymer such that the relative distances between them and the edges of the Polycomb-repressed gene were the same as in the *inv-en* locus (Fig. 7*C*) and performed the MC-MD simulations in the same way as described in previous sections. Measurements of the distances between each PRE and other monomers (Fig. 7*D*) or the heat-map of the pairwise distances between monomers (Fig. 7*E*) indicate that the simulated PREs cluster. Overall, we conclude that looping interactions between PRE-anchored PRC1 and tri-methylated H3K27 automatically increase the likelihood that PREs are found in closer proximity compared to other monomers located at similar linear distances.

## Discussion

Two main conclusions follow from the observations presented here. First, in the milieu of *Drosophila* nuclei, the stochastic interactions of the PRE-anchored PRC1 with tri-methylated H3K27 are sufficient to fold the methylated chromatin. This effectively translates the epigenetic marking of the Polycomb-repressed genes into chromatin folding. Conceivably, such folding competes with processes required for transcriptional activity, for example, chromatin looping required for enhancer-promoter interactions. Since the extent of the folding depends on the affinities of individual PREs to PRC1 and their relative arrangement, changes of either or both will allow the evolutionary selection of combinations tailored for the regulation of specific genes. Second, the chromatin folding by PRE-anchored PRC1 leads to spatial clustering of the DNA elements to which PRC1 is bound. Remarkably, the clustering does not require specific protein-protein interactions. It emerges during chromatin folding and depends on the relative positions of PREs inside the Polycomb-repressed genes. Such clustering may be reinforced by interactions between PRC1 complexes (30, 31, 32). Processes other than PRC1-H3K27me3 interactions also affect chromatin topology, for example, transcription or chromatin insulators. Not considered here, these processes may need to be taken into account to fully describe the chromatin folding at specific loci repressed by the Polycomb system.

We note that probabilistic clustering does not depend on the molecular nature of the anchor points or chromatin "stickiness". For example, enhancer elements bound by proteins that interact with elongating RNA polymerase complexes may perceive intensively transcribed genes as stretches of "sticky" chromatin. This, in turn, will lead to enhancer clustering. Decades of prior experimental studies gave us the opportunity to calibrate our Monte Carlo – Molecular Dynamics simulations such that they yielded a reasonably realistic representation of the *Drosophila* Polycomb-repressed gene. While very few other epigenetic systems are investigated in comparable detail, as our understanding of these systems grows, the MC-MD approach presented here will likely be useful to explore them.

## Experimental procedures

### Polymer model

We describe chromatin using the standard biopolymer model from (33). The model builds on three types of interactions associated with stretching, bending, and excluded volume preventing two monomers from occupying the same space. We review each of these contributions below.

#### Stretching—finitely extensible spring

We model stretching between two neighboring monomers with the non-linear Warner spring (34), also known as the FENE potential:

(1) $$V_{FENE}\left(r\right)=\left\{ \begin{array}{lll} -\frac{1}{2}K_{s}{\left(\frac{R_{0}}{\sigma }\right)}^{2}\ln\left[1-{\left(\frac{r}{R_{0}}\right)}^{2}\right] & & r\leq R_{0}\\ \infty & otherwise & \end{array}\right.$$

Here, r denotes the absolute distance between two monomer centers, $K_{s}$ is the potential’s strength, $R_{0}$ is the spring’s maximum extension, and σ is the nucleosome diameter (σ=10 nm). We use $R_{0}=1.5\sigma$ (35) and $K_{s}=30\;k_{B}T$, where $k_{B}$ is Boltzmann’s constant and T is absolute temperature, which gives the average bond length ≈0.97σ.

#### Bending—polymer stiffness

To describe the stiffness, we use a bending potential that is proportional to the cosine of the angle θ between neighboring bonds (*i.e.* the angle between next-nearest-neigbor monomers)

(2) $$V_{bend}\left(\theta \right)=K_{\theta }\left(1-\cos\left(\theta \right)\right)$$

We set the bending constant to $K_{\theta }=3k_{B}T$. This corresponds to the persistence length $l_{p}=K_{\theta }\sigma /\left(k_{B}T\right)=3\sigma =30$ nm (36). In our simulations, we have on average $l_{p}\approx 2.7\sigma$.

#### Excluded volume—no monomer–monomer overlap

**Figure 8:**
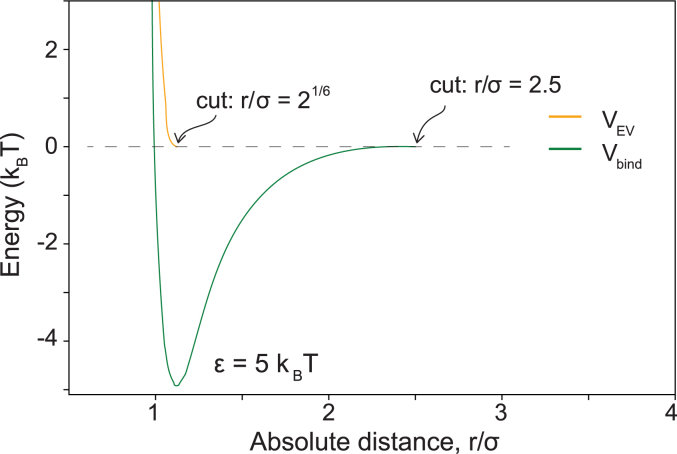
**Attraction and repulsion potentials in the polymer model.** The curves show the binding energy $V_{bind}\left(r\right)$ (*green*) and the excluded-volume repulsion energy $V_{EV}\left(r\right)$ (*orange*) when $\varepsilon =5k_{B}T$. We rescaled the x-axis with the nucleosome diameter σ=10 nm.

We model excluded volume interactions between two monomers from a truncated version of the Lennard-Jones potential

(3) $$V_{LJ}\left(r\right)=4\varepsilon \left[{\left(\frac{\sigma }{r}\right)}^{12}-{\left(\frac{\sigma }{r}\right)}^{6}\right]$$

where, ε≥0 is the potential’s strength and, as before, σ is the monomer diameter and r the absolute distance. However, as $V_{LJ}$ includes both repulsion and attraction, we turn it into an excluded volume interaction, $V_{EV}$, by cutting the potential at the minimum ($r=2^{1/6}\sigma$) and shifting it upwards by ε. For distances beyond the cut, the interaction energy is zero (Fig. 8, yellow curve). In other words,

(4) $$V_{EV}\left(r\right)=\left\{ \begin{array}{ll} V_{LJ}\left(r\right)+\varepsilon & r\leq 2^{1/6}\sigma \\ 0 & otherwise. \end{array}\right.$$

We put $\varepsilon =1\;k_{B}T$ to get a strong enough repulsion.

#### Monomer–monomer attraction

In addition to the standard polymer properties—stretching, bending, and excluded volume interactions—some monomers also attract each other. Similar to (35), we model such attraction using the Lennard-Jones potential (Equation 3) and assume that the interactions do not extend beyond some interaction distance $r=r_{\mathrm{int}}$, after which the binding potential is zero. In summary, we model monomer attractions with

(5) $$V_{bind}\left(r\right)=\left\{ \begin{array}{ll} V_{LJ}\left(r\right)-V_{LJ}\left(r_{\mathrm{int}}\right), & r\leq r_{\mathrm{int}}\\ 0, & otherwise \end{array}\right.$$

where $r_{\mathrm{int}}=2.5\sigma$. We show $V_{EV}\left(r\right)$ and $V_{bind}\left(r\right)$ in Figure 8 for easy comparison.

#### Equation of motion

Just as in (33), we assume that each monomer undergoes Brownian motion and subject to a force F associated with the interaction potentials specified above. Denoting $r_{i}$ as the i th monomer’s 3D coordinate and $V\left(r_{i}\right)$ as the total interaction potential (sum of Equations (1), (2), (3), (4), (5)), the force is $F_{i}=-\nabla V\left(r_{i}\right)$. We use the Langevin equation for the monomers’ equation of motion.

(6) $$m\frac{d^{2}r_{i}\left(t\right)}{dt^{2}}=-\Gamma \frac{dr_{i}\left(t\right)}{dt}+F_{i}+W_{i}\left(t\right)$$

where Γ is the damping constant coupling the monomers to the surrounding fluid and $W_{i}\left(t\right)$ is a white-noise force term. The noise’s amplitude is related to Γ *via* the diffusion constant D, using the Einstein relation $D=k_{B}T/\Gamma$. We simulate Equation 6 using LAMMPS.

### Polymer configuration and monomer types

We simulate a polymer consisting of 360 monomers. As each monomer represents a nucleosome, the whole polymer corresponds to approximately a 63 kb chromatin segment. The polymer is divided into three sections (see Fig. 1*A*). The middle third contains H3K27me3 nucleosomes (green) with a few interspersed PREs (grey or orange). The two other thirds hold only unmethylated monomers (red).

PREs come in two flavors: PRC1-bound (orange) or unbound (grey). If bound, they attract methylated monomers and can form long-lived loops if appearing close enough in 3D. We do not consider the attraction between PRC1-bound PREs and unmethylated monomers (red). Before the polymer simulation starts, we assign PRC1s randomly to the PREs (Fig. 1*B*). We outline this procedure in the section devoted to the Monte Carlo - Molecular Dynamics framework.

We adjusted the flanking regions’ length (and the volume of the simulation box) to calibrate the monomer volume fraction to the nucleosome density in the nucleus. In our simulation, we use 360 monomers, each having size σ=10 nm, residing in a total volume that is ${\left(24\sigma \right)}^{3}$ large. This yields the volume fraction $360/24^{3}$ = 0.026. This is very close to the nuclear volume fraction that we estimates as follows. First, the volume of *Drosophila*’s cell nucleus is 78 μ m^3^. It holds approximately 1.65 million nucleosomes (the total DNA length is $2.88\cdot 10^{8}$ bp and each nucelsome contains 175 bp). Second, partitioning the nuclear volume into nucleosomes-sized 10 nm subvolumes, each having volume $10^{-6}\mu$ m^3^, there are $78\cdot 10^{6}$ of such volumes. Given 1.65 million nucleosomes, the filling fraction is $1.65\cdot 10^{6}/78\cdot 10^{6}=0.021$.

### Monte Carlo–molecular dynamics scheme

Instead of simulating diffusing PRC1 proteins surrounding the polymer searching for target monomers, we use binding probabilities to assign PRC1s randomly to the PREs prior to the polymer simulation. We start by making the assignment, and then introduce interaction potentials and simulate the polymer’s motion in LAMMPS. Below, we outline the main steps in our numerical approach.

1) Set binding probability $p_{b}^{PRE}$
2) Sequentially go through all monomers i=1,...,360. For each monomer:
  a) Draw a random number r uniformly distributed between 0 and 1.
  b) Populate the monomers with PRC1s based on r and the binding probabilities. We consider two cases:
    i) If the i th monomer is a PRE and $r<p_{b}^{PRE}$, we introduce the binding potential $V_{b}$ (Equation 5) between the PRE and all methylated monomers.
    ii) If the i th monomer is a methylated or unmehtylated nucleosome, we leave it unbound. In other words, we assume that $p_{b}^{me3}=p_{b}^{unmeth.}=0$. However, we point out that this choice is not a limitation of our framework and nothing prevents us from considering non-zero binding probabilities and introducing weak interactions potentials.
3) Run polymer simulations. After we assigned PRE with PRC1s, we simulate the polymer in LAMMPS. At regular time intervals, we store the monomers’ 3D coordinates that we use to calculate averages. We stop the simulation after 100 s ($∼10^{9}$ LAMMPS MD steps) when we consider the PRC1s as unbound.

We point out that we calculate averages over uncorrelated polymer snapshots and repeated PRE assignments keeping $p_{b}^{PRE}$ constant. That is, first we set $p_{b}^{PRE}$, then we collect data as we cycle through the above items 2 to 3 several times, and then we calculate averages.

### Simulation details: system preparation, equilibration, and simulation time

To study the polymer fluctuations, we used the LAMMPS molecular dynamics package. We included interaction potentials specified in Equations (1), (2), (3), (4), (5) and then simulated the polymer under fixed temperature and volume conditions with periodic boundary conditions. Furthermore, we used a Langevin thermostat to treat the surrounding water as an implicit solvent and integrated the monomers’ equation of motion (Equation 6) using a velocity Verlet algorithm. Below, we outline critical steps in our simulation scheme.

#### Simulation time scales

We integrate Equation 6 using the velocity Verlet algorithm. Like in (35), we set the MD timescale τ by considering the diffusion constant D. Using $D=5\;{\left(\mu m\right)}^{2}s^{-1}$ (reasonable for a 10 nm macromolecule) gives

(7) $$\tau =\frac{\sigma^{2}}{D}=\frac{{\left(10\;nm\right)}^{2}}{5\frac{{\left(\mu m\right)}^{2}}{s}}=20\mu s$$

In terms of τ, we set damping term and integration step to $\Gamma =0.5\tau^{-1}$ and Δt=0.012τ.

#### System preparation

Before starting the simulation, we remove all monomer-monomer overlaps. This step ensures that force gradients do not diverge, causing the simulation to crash. We achieve this in three steps. First, we randomly place the monomers in a giant simulation box, much bigger than we use later on. Second, we push overlapping monomers apart using the soft potential

(8) $$\varphi =A\left[1+\cos\left(\frac{\pi r}{r_{c}}\right)\right],r<r_{c},\varphi =0,otherwise$$

where $r_{c}=2.5\sigma$, and where we increased A from 0 to 40 in linear increments during a short MD run (just a few MD steps). In the third step, we remove φ and make a quick run (about 500 MD steps) to reduce the volume and achieve the desired monomer density (and pressure). After these steps, we have a non-overlapping polymer inside the target volume.

#### Equilibration

After achieving the desired density, we let the polymer relax to an equilibrated state with respect to the interaction potentials we specified in Equations (1), (2), (3), (4) (excluding monomer-monomer attractions). The equilibrium process takes $2\times 10^{6}$ MD steps under fixed volume and temperature conditions, and periodic boundaries. The simulation time is long enough so that the polymer’s center-of-mass starts to diffuse, indicating that all internal degrees of freedom are equilibrated, such as single monomer fluctuations.

#### Simulation time

After completing the equilibration step, we populate PREs with PRC1 complexes as described above and introduce positive attractions accordingly using Equation 5. Then we simulate the polymer’s fluctuations and sample its 3D configuration at regular (non-correlated) time intervals. We stop the simulation after $100\times 10^{6}$ MD steps, corresponding to 100 s, which is associated with typical PRC1 residence times (18).

#### Equipment

We ran all our simulations on the High Performance Computing Center North (HPC2N, www.hpc2n.umu.se) a center for Scientific and Parallel Computing. We used ≲5 computer nodes each having 128 GB of memory and 2×14 cores (CPU: Intel Xeon E5-2690v4). Depending on binding strengths, the polymer equilibration took ∼3−7 days. The ensuing simulation where we store the polymer’s 3D coordinates, took ∼1−3 days.

### Volume and the Radius of Gyration

To understand by how much the methylated polymer region folds as we change $p_{b}^{PRE}$, we calculated the region’s volume V using the Radius of Gyration $R_{G}$, where $V=\frac{4}{3}\pi R_{G}^{3}$. For a polymer with N segments, $R_{G}$ is the average distance between each monomer and the polymers’ center point $r_{c}=\frac{1}{N}\sum_{k=1}^{N}r_{k}$. That is,

(9) $$R_{G}^{2}=\frac{1}{N}\sum_{k=1}^{N}{\left(r_{k}-r_{c}\right)}^{2}$$

As we only calculate $R_{G}$ for monomers inside the methylated regions, we restrict the monomer indices to i=120,...,240.

### Derivation of the PRC1-PRE binding probability $p_{b}^{PRE}$

Here we will derive Equation 21 in the main text. First, assuming first order kinetics, we have

(10) $$PRC1+PRE↔\mathrm{PR}E^{\prime }$$

where $\mathrm{PR}E^{\prime }$ denotes PRC1-bound PREs. In equilibrium, we have

(11) $$K_{D}^{PRE}=\frac{c_{PRC1}c_{PRE}}{c_{\mathrm{PR}E^{\prime }}}$$

where $c_{X}$ denotes concentration. To get $p_{b}^{PRE}$, we calculate the fraction of bound PREs, that is

(12) $$p_{b}^{PRE}=\frac{c_{\mathrm{PR}E^{\prime }}}{c_{PRE}^{tot}}$$

where $c_{PRE}^{tot}=c_{\mathrm{PR}E^{\prime }}+c_{PRE}$ (total PRE concentration). Using $c_{PRE}^{tot}$, $p_{b}^{PRE}$, and the definition for $K_{D}^{PRE}$ [Equation 11], we obtain Equation 21 in the Results section:

(13) $$\begin{array}{rrr} p_{b}^{PRE} & = & \frac{c_{\mathrm{PR}E^{\prime }}}{c_{\mathrm{PR}E^{\prime }}+c_{PRE}}=\frac{1}{1+\frac{K_{D}^{PRE}}{c_{PRC1}}}\\ & = & \frac{c_{PRC1}}{c_{PRC1}+K_{D}^{PRE^{\prime }}} \end{array}$$

## Data availability

All data required to reproduce the results is included in the manuscript.

## Supporting information

This article contains supporting information.

## Conflict of interest

The authors declare that they have no conflicts of interest with the contents of this article.

## References

[1] Piunti A., Shilatifard A. (2021). The roles of Polycomb repressive complexes in mammalian development and cancer. Nat. Rev. Mol. Cell Biol..

[2] Schuettengruber B., Bourbon H.M., Di Croce L., Cavalli G. (2017). Genome regulation by polycomb and Trithorax: 70 years and counting. Cell.

[3] Czermin B., Melfi R., McCabe D., Seitz V., Imhof A., Pirrotta V. (2002). Drosophila enhancer of Zeste/ESC complexes have a histone H3 methyltransferase activity that marks chromosomal Polycomb sites. Cell.

[4] Muller J., Hart C.M., Francis N.J., Vargas M.L., Sengupta A., Wild B. (2002). Histone methyltransferase activity of a Drosophila Polycomb group repressor complex. Cell.

[5] Fischle W., Wang Y., Jacobs S.A., Kim Y., Allis C.D., Khorasanizadeh S. (2003). Molecular basis for the discrimination of repressive methyl-lysine marks in histone H3 by Polycomb and HP1 chromodomains. Genes Dev..

[6] Pengelly A.R., Copur O., Jackle H., Herzig A., Muller J. (2013). A histone mutant reproduces the phenotype caused by loss of histone-modifying factor Polycomb. Science.

[7] Coleman R.T., Struhl G. (2017). Causal role for inheritance of H3K27me3 in maintaining the OFF state of a Drosophila HOX gene. Science.

[8] Laprell F., Finkl K., Muller J. (2017). Propagation of Polycomb-repressed chromatin requires sequence-specific recruitment to DNA. Science.

[9] Boettiger A.N., Bintu B., Moffitt J.R., Wang S., Beliveau B.J., Fudenberg G. (2016). Super-resolution imaging reveals distinct chromatin folding for different epigenetic states. Nature.

[10] Kassis J.A., Brown J.L. (2013). Polycomb group response elements in Drosophila and vertebrates. Adv. Genet..

[11] Kahn T.G., Schwartz Y.B., Dellino G.I., Pirrotta V. (2006). Polycomb complexes and the propagation of the methylation mark at the Drosophila ubx gene. J. Biol. Chem..

[12] Papp B., Muller J. (2006). Histone trimethylation and the maintenance of transcriptional ON and OFF states by trxG and PcG proteins. Genes Dev..

[13] Schwartz Y.B., Kahn T.G., Nix D.A., Li X.Y., Bourgon R., Biggin M. (2006). Genome-wide analysis of Polycomb targets in Drosophila melanogaster. Nat. Genet..

[14] Kahn T.G., Dorafshan E., Schultheis D., Zare A., Stenberg P., Reim I. (2016). Interdependence of PRC1 and PRC2 for recruitment to polycomb response elements. Nucleic Acids Res..

[15] Blackledge N.P., Klose R.J. (2021). The molecular principles of gene regulation by Polycomb repressive complexes. Nat. Rev. Mol. Cell Biol..

[16] Kang H., McElroy K.A., Jung Y.L., Alekseyenko A.A., Zee B.M., Park P.J. (2015). Sex comb on midleg (Scm) is a functional link between PcG-repressive complexes in Drosophila. Genes Dev..

[17] Plimpton S. (1995). Fast parallel algorithms for short-range molecular-dynamics. J. Comput. Phys..

[18] Ficz G., Heintzmann R., Arndt-Jovin D.J. (2005). Polycomb group protein complexes exchange rapidly in living Drosophila. Development.

[19] Phillips R., Kondev J., Theriot J., Garcia H. (2013).

[20] Bonnet J., Lindeboom R.G.H., Pokrovsky D., Stricker G., Çelik M.H., Rupp R.A.W. (2019). Quantification of proteins and histone marks in Drosophila Embryos reveals Stoichiometric relationships Impacting chromatin regulation. Dev. Cell.

[21] Schwartz Y.B., Kahn T.G., Stenberg P., Ohno K., Bourgon R., Pirrotta V. (2010). Alternative epigenetic chromatin states of polycomb target genes. PLoS Genet..

[22] Steffen P.A., Fonseca J.P., Ganger C., Dworschak E., Kockmann T., Beisel C. (2013). Quantitative *in vivo* analysis of chromatin binding of Polycomb and Trithorax group proteins reveals retention of ASH1 on mitotic chromatin. Nucleic Acids Res..

[23] Bernstein E., Duncan E.M., Masui O., Gil J., Heard E., Allis C.D. (2006). Mouse polycomb proteins bind differentially to methylated histone H3 and RNA and are enriched in facultative heterochromatin. Mol. Cell. Biol..

[24] Kaustov L., Ouyang H., Amaya M., Lemak A., Nady N., Duan S. (2011). Recognition and specificity determinants of the human cbx chromodomains. J. Biol. Chem..

[25] Barbieri M., Chotalia M., Fraser J., Lavitas L.M., Dostie J., Pombo A. (2012). Complexity of chromatin folding is captured by the strings and binders switch model. Proc. Natl. Acad. Sci. U. S. A..

[26] Petryk N., Dalby M., Wenger A., Stromme C.B., Strandsby A., Andersson R. (2018). MCM2 promotes symmetric inheritance of modified histones during DNA replication. Science.

[27] Yu C., Gan H., Serra-Cardona A., Zhang L., Gan S., Sharma S. (2018). A mechanism for preventing asymmetric histone segregation onto replicating DNA strands. Science.

[28] Eagen K.P., Aiden E.L., Kornberg R.D. (2017). Polycomb-mediated chromatin loops revealed by a subkilobase-resolution chromatin interaction map. Proc. Natl. Acad. Sci. U. S. A..

[29] Ogiyama Y., Schuettengruber B., Papadopoulos G.L., Chang J.M., Cavalli G. (2018). Polycomb-dependent chromatin looping contributes to gene Silencing during Drosophila development. Mol. Cell.

[30] Gambetta M.C., Muller J. (2014). O-GlcNAcylation prevents aggregation of the Polycomb group repressor polyhomeotic. Dev. Cell.

[31] Kim C.A., Gingery M., Pilpa R.M., Bowie J.U. (2002). The SAM domain of polyhomeotic forms a helical polymer. Nat. Struct. Biol..

[32] Kim C.A., Sawaya M.R., Cascio D., Kim W., Bowie J.U. (2005). Structural organization of a Sex-comb-on-midleg/polyhomeotic copolymer. J. Biol. Chem..

[33] Kremer K., Grest G.S. (1990). Dynamics of entangled linear polymer Melts - a molecular-dynamics simulation. J. Chem. Phys..

[34] Warner H.R. (1972). Kinetic-theory and rheology of dilute suspensions of finitely extendible dumbbells. Ind. Eng. Chem. Fund..

[35] Annunziatella C., Chiariello A.M., Bianco S., Nicodemi M. (2016). Polymer models of the hierarchical folding of the Hox-B chromosomal locus. Phys. Rev. E.

[36] Langowski J. (2006). Polymer chain models of DNA and chromatin. Eur. Phys. J. E Soft Matter.

