# Supplementary material for: Polycomb proteins translate histone methylation to chromatin folding
Source: J Biol Chem. 2023 Jul 25;299(9):105080. doi: 10.1016/j.jbc.2023.105080 (PMC10470199; doi:10.1016/j.jbc.2023.105080)
Supplement: Supporting Figure S1 [file mmc1.pdf]

Polycomb proteins translate histone methylation to chromatin folding

Ludvig Lizana[1,*], Negar Nahali[1,2], Yuri B. Schwartz[3,*]

[1] Department of Physics, Integrated Science Lab, Umeå University, SE-901 87 Umeå, Sweden.

[2] Centre for bioinformatics, Department of Informatics, University of Oslo, NO-0373 Oslo, Norway

[3] Department of Molecular Biology, Umeå University, SE-901 87 Umeå, Sweden

Supplementary materials

Figure S1

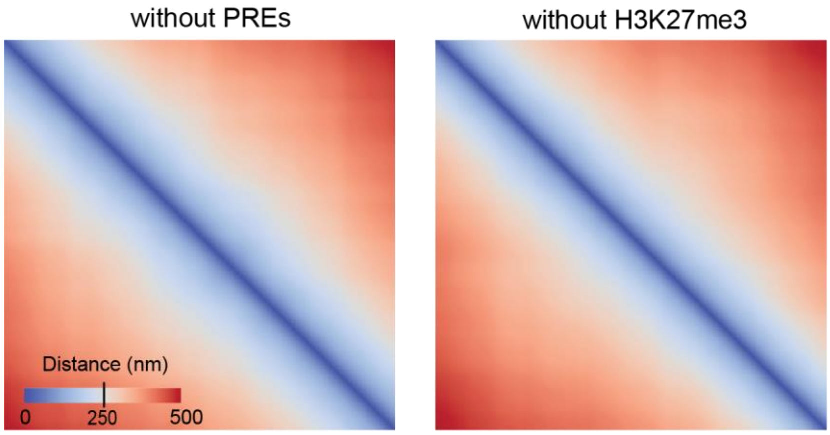

Figure S1. Chromatin folding in the absence of PREs or H3K27me3. Heat-map representations of pairwise distances between monomers show that the central part of the polymer does not fold if it has methylated nucleosomes but lacks PREs (left panel) or if it contains PREs but lacks methylated nucleosomes (right panel).
